# Spatial and demographic patterns of Cholera in Ashanti region - Ghana

**DOI:** 10.1186/1476-072X-7-44

**Published:** 2008-08-12

**Authors:** Frank B Osei, Alfred A Duker

**Affiliations:** 1 Department of Geomatic Engineering, Kwame Nkrumah University of Science and Technology (KNUST), Kumasi, Ghana

## Abstract

**Background:**

Cholera has claimed many lives throughout history and it continues to be a global threat, especially in countries in Africa. The disease is listed as one of three internationally quarantinable diseases by the World Health organization, along with plague and yellow fever. Between 1999 and 2005, Africa alone accounted for about 90% of over 1 million reported cholera cases worldwide. In Ghana, there have been over 27000 reported cases since 1999. In one of the affected regions in Ghana, Ashanti region, massive outbreaks and high incidences of cholera have predominated in urban and overcrowded communities.

**Results:**

A GIS based spatial analysis and statistical analysis, carried out to determine clustering of cholera, showed that high cholera rates are clustered around Kumasi Metropolis (the central part of the region), with Moran's Index = 0.271 and *P *< 0.001. Furthermore, A Mantel-Haenszel *Chi square *for trend analysis reflected a direct spatial relationship between cholera and urbanization (*χ*^2 ^= 2995.5, *P *< 0.0001), overcrowding (*χ*^2 ^= 1757.2, *P *< 0.0001), and an inverse relationship between cholera and order of neighborhood with Kumasi Metropolis (*χ*^2 ^= 831.38, *P *< 0.0001).

**Conclusion:**

The results suggest that high urbanization, high overcrowding, and neighborhood with Kumasi Metropolis are the most important predictors of cholera in Ashanti region.

## Background

Cholera has claimed many lives throughout history and it continues to be a global threat [1], especially in countries in Africa. Between 1999 and 2005, there were over 1 million reported cholera cases and over 28,000 reported deaths worldwide. Africa alone accounted for about 90% of the cases and 96% of the deaths worldwide [2-8]. Cholera has gained both global and public health attention due to its mode of transmission and severity. For instance it has become one of the most researched communicable diseases. The disease is also listed as one of three internationally quarantinable diseases by the World Health organization (WHO), along with plague and yellow fever [9]. In addition to human suffering and lives loss, cholera outbreak causes panic, disrupts the social and economic structure and can impede development in the affected communities [6].

Cholera reached West Africa and Ghana during the seventh pandemic [10-13]. The disease has been endemic in Ghana since its introduction in the 1970's [14]. From 1999 to 2005, a total of 26,924 cases and 620 deaths were officially reported to the WHO [2-8]. Although the disease is transmitted mainly through contaminated water and food, several demographic and geographic factors can predispose an individual or groups of individuals to infection. For example, increase in population density can strain existing sanitation systems, thus putting people at increased risk of contracting cholera [15,16]. Once the bacterial, *V. cholerae*, are present in water in sufficient dose, an outbreak can trigger and propagate depending on demographic factors such as population density [17,18], urbanization, and overcrowding [19]. In developing countries like Ghana, high incidence of cholera seems to predominate in the urban communities, and this is primarily due to high overcrowding and unsanitary living conditions in urban communities. While cholera is prevalent in low urban communities in certain geographical areas like Mexico [19], the disease has predominated in urban and overcrowded communities in Ghana. Intermittent water supply coupled with indiscriminate sanitation practices in urban communities in Ghana puts inhabitants at risk of contracting cholera.

Studies on diarrhea related diseases in Ghana [20] so far have focused solely on the biological factors and characteristics of the individuals affected. Although such studies are very useful, they omit the spatial and regional variations of the critical risk factors. Such studies also fail to define territories at high risk. Since health levels vary substantially between different regions, it is essential to characterize these regional variations and identify areas with an accumulation of health problems for epidemiological research, and to allow appropriate public health policy decisions [21,22]. Advances in Geographical Information Systems (GIS) technology provide new opportunities for environmental epidemiologist to study associations between demographic and environmental exposures and the spatial distribution of diseases [23]. GIS has been used in the surveillance and monitoring of vector-borne diseases [24,25], water-borne diseases [26], in environmental health [27-29], analysis of disease policy and planning [30]. Several cholera studies [17-19,31-34] have also employed GIS technologies. This study focuses on the application of a GIS based spatial analyses and statistical technology to study the spatial patterns of cholera, identify territories of high risk, and determine demographic risk factors that contribute to high rates of cholera. No study so far has looked at the spatial patterns of cholera in Ghana. There is therefore no information and/or knowledge about its spatial patterns and demographic correlates in Ghana. Studying the spatial and demographic patterns of cholera in Ghana will prove useful for health officials and policymakers to make appropriate planning and resource allocation.

## Results and analyses

**Figure 2:**
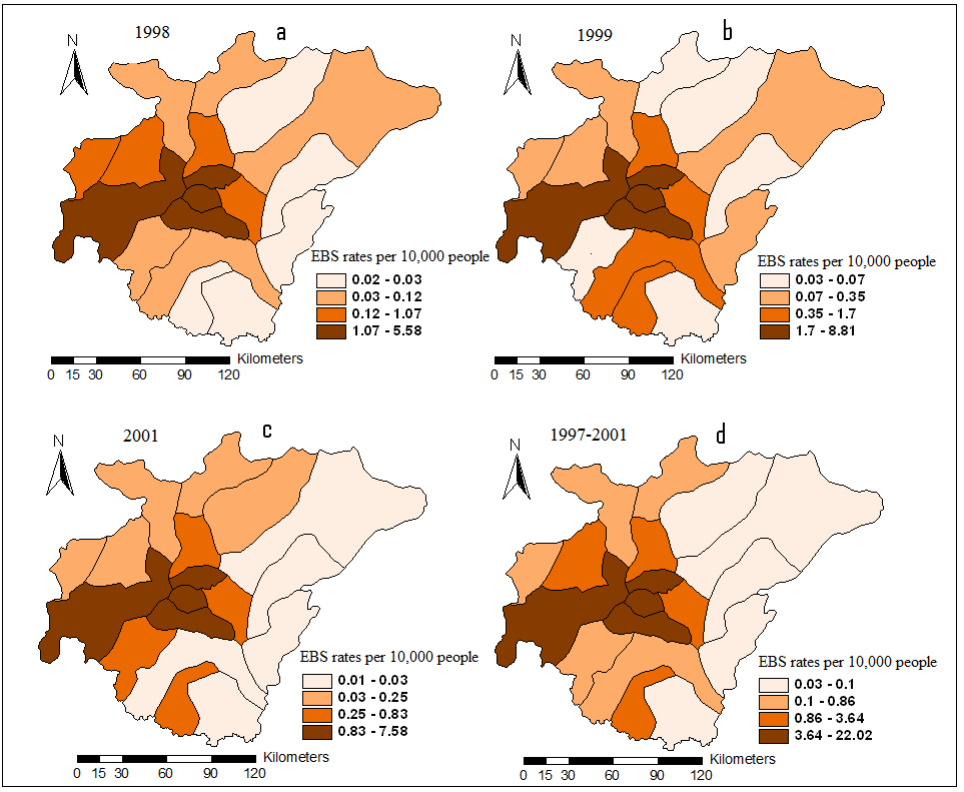
**EBS Smoothed Rates of Cholera for 1998 (2a), 1999(2b), 2001(2c), and 1997–2001(2d)**.

The extent to which neighboring values are correlated was measured using Global Moran's index. A significance assessment through a permutation procedure was implemented to determine the significance of the computed Moran's index. There is a positive and statistically significant spatial autocorrelation for cumulative incidence rate of cholera from 1997 to 2001 (Moran's I = 0.271, *P-value *= 0.0009). Moreover, a spatial autocorrelation statistic computed for each of the periods 1998, 1999 and 2001 were statistically significant (*P-value *< 0.05) for Moran's I (See Table 1). This reflects clustering of high rates of cholera at the central part of the region (See Figure 2).

**Table 1:** Moran's I for spatial autocorrelation computed for cumulative incidence of cholera (1997–2001), and the specific years of cholera outbreaks (1998, 1999, and 2001).

**Year**	**Moran's I**	***P*-value**
1997–2001	0.271	0.0001
1998	0.331	0.004
1999	0.181	0.040
2001	0.240	0.010

Figure 2 also shows the Empirical Bayesian smoothed rates of cholera. A visual inspection reflects clustering of high rates of cholera at areas surrounding Kumasi Metropolis and its adjoining neighbors (See Figure 2d). Moreover, clustering of high rates of cholera was persistent at Kumasi Metropolis and its adjoining neighbors in the years 1998, 1999, and 2001 (See Figures 2a, 2b, 2c).

The rate ratio within each stratum was computed using EpiInfo, and the results shown in Tables 2, 3, 4, 5, 6. The cumulative incidence rate of cholera was 22 times higher in the first order neighborhood stratum than the third order neighborhood stratum. The cumulative incidence rate in the second order neighborhood (with Kumasi Metropolis) was not very high (1.4 times) compared to the third order neighborhood (See Table 2). Cholera incidence rate in the most urban stratum was about 20 times higher than the least urban stratum (Table 3), while the incidence rate in the most overcrowded stratum was about 30 times higher than the less overcrowded stratum (Table 4). A *Chi square *for trend analysis reflected a direct spatial relationship between cholera and urbanization (Table 3: *χ*^2 ^= 2995.5, *P *= 0.000001), overcrowding (Table 4: *χ*^2 ^= 1757.2, *P *= 0.000001), and an inverse relationship between cholera and order of neighborhood (Table 2: *χ*^2 ^= 831.38, *P *= 0.000001).

**Table 2:** Cholera incidence rate and population-based rate ratios by strata of districts classified according to order of neighborhood and/or adjacency to Kumasi Metropolis, 1997–2001.

**Order of neighborhood**	**Cholera** ** cases**	**Sub** ** Population**	**Rate (per 10000)**	**Rate ratio (95%CI)**
**First order**	562	672482	8.357101008	22 (14.14–35.27)
**Second order**	69	1307843	0.527586262	1.4 (0.84–2.36)
**Third order**	21	558356	0.376104134	Reference

Chi-square for linear trend = 831.375, P-value = 0.000001

**Table 3:** Cholera incidence rate and population-based rate ratios by strata of districts classified according to level of urbanization, 1997–2001.

**Urbanization (%)**	**Cholera** ** cases**	**Sub** ** Population**	**Rate (per 10000)**	**Rate ratio(95%CI)**
**Low**				
0.0–16.5	126	1065586	1.182447968	Reference
**Medium**				
19.2–35.6	357	848903	4.205427475	3.56 (2.89–4.38)
**High**				
38.9–100	2748	1170270	23.48176062	19.86 (16.55–23.840

Chi-square for linear trend = 2995.5, P-value = 0.000001

**Table 4:** Cholera incidence rate and population-based rate ratios by strata of districts classified according to level of overcrowding, 1997–2001.

**Overcrowding index**	**Cholera** ** cases**	**Sub** ** Population**	**Rate (per 10000)**	**Rate ratio(95%CI)**
**Low**				
(-4.5 < OI < -1.66)	51	1048225	0.49	Reference
**Medium**				
(-0.73 < OI < -0.37)	310	764556	4.05	8.33 (6.14–11.34)
**High**				
(-0.25 < OI < 3.20)	2870	1896170	15.14	31.11 (23.40–41.46)

Chi-square for linear trend = 1757.2, P-value = 0.000001

**Table 5:** Cholera incidence rate and population-based rate ratios by strata of districts classified according to urbanization level and order of neighborhood and/or adjacency to Kumasi Metropolis, 1997–2001.

**Urbanization (%)**	**Neighborhood**	**Cholera cases**	**Population**	**Rate(per10000)**	**Rate ratio(95% CI)**
**Low**	Adjoining	84	146028	5.75	12.59 (8.57–18.55)
0.0–16.5	Non-adjoining	42	919558	0.46	Reference
					
**Medium**	Adjoining	330	361786	9.12	16.46 (10.96–24.88)
19.2–35.6	Non-adjoining	27	487117	0.55	Reference
					
**High**	Adjoining	148	164668	8.99	19.67 (12.22–31.95)
38.9–100	Non-adjoining	21	459524	0.46	Reference

**Table 6:** Cholera incidence rate and population-based rate ratios by strata of districts classified according to overcrowding level and order of neighborhood and/or adjacency to Kumasi Metropolis, 1997–2001.

**Overcrowding**	**Neighborhood**	**Cholera cases**	**Population**	**Rate(per 10,000)**	**Rate ratio(95% CI)**
**Low**	Adjoining	0	0	0.00	0
(-4.5 < OI < -1.66)	Non-adjoining	51	1048225	0.49	*
					
**Medium**	Adjoining	286	237610	12.04	26.43 (17.16–41.05)
(-0.73 < OI < -0.37)	Non-adjoining	24	526946	0.46	Reference
					
**High**	Adjoining	276	434872	6.35	12.31 (7.17–21.51)
(-0.25 < OI < 3.20)	Non-adjoining	15	291028	0.52	Reference

*undefined

The cumulative incidence rate of cholera was higher in the adjoining (first order neighborhood) stratum than in the non-adjoining stratum (second and third order neighborhoods) within each urbanization stratum (Table 5). Similar pattern was observed in the high and medium overcrowding strata (Table 6). No adjoining district was within the least overcrowded stratum.

## Conclusion

This study has demonstrated the use of spatial statistical analysis and GIS to map hotspots, and the spatial dependency of cholera distribution within a population. Through spatial statistical procedures, non-randomness in the distribution of cholera and the identification of unusual concentration of cholera incidence has been defined. This therefore prompts health planners in the country to take a critical look at these risk areas, and make appropriate health planning and resource allocation. In conclusion, the results of this research suggest that high urbanization, high overcrowding, and neighborhood with Kumasi Metropolis are the most important predictors of cholera in Ashanti region-Ghana. It is therefore necessary that health officials and policy makers reasonably improve their surveillance systems to prepare for the possibility of sustained transmission should an infection be introduced. Since this research is the first of its kind in Ghana, a more detailed research is required to consider factors like access to safe drinking water, and availability of waste disposal systems to thoroughly evaluate the risk of cholera in the region.

## Materials and methods

### Research methodology

An important part of health-needs assessment is the identification of high risk areas for a disease because understanding the characteristics of high risk areas may indicate what is needed to improve health care provision [35]. Several disease clustering techniques have been developed to define territories of high risk [36-38]. However, using clustering detection technique to define high risk territories is only an exploratory technique to locate clusters, but does not establish a relation between the disease and risk factors. In this study, Moran's I for spatial autocorrelation was computed to ascertain evidence of cholera clustering. A global Bayesian smoothing technique was employed to smooth the crude rates of cholera, and then mapped to determine the spatial distribution of cholera. The districts in the region were classified into strata of districts based on demographic indicators. Population-based incidence rate ratios were then computed for each stratum to determine territories of high risk. The Extended Mantel-Haenszel *Chi Square *for trend analyses and associated *P values *(one degree of freedom) were also computed to determine the trend between the demographic factors and *V. cholerae *infection [39].

### The study area

#### History of Cholera in Ghana

On 1^st ^September, 1970 a Togolese national in transit at the Kotoka International Airport from Conakry, Guinea, collapsed and was found to have cholera [14]. This was the announcement of the arrival of the seventh pandemic of cholera in Ghana. However, an outbreak did not begin from then until it was smuggled into the country through fishing [40]. At that time, some Ghanaians went for fishing in the waters of Togo, Liberia and Guinea. One of the fishermen died and although a sanitary cordon had been placed on our borders, his family smuggled the corpse into his home town, and the usual burial rites were performed. It was after this that cholera began to spread along the shores of Ghana. The disease swept through many coastal villages in epidemic proportions. It kept on spreading and by July 1971, Ashanti region began to report cases, indicating that cholera was spreading across the country [40]. During those periods, reported outbreaks were investigated, treatment camps were set, people were vaccinated against cholera, and the population was also educated on measures to prevent the spread of the disease. However, all these attempts to prevent cholera from taking root in Ghana failed. Since then cholera has existed in both epidemic and endemic forms in Ghana.

#### Location and demography of the study area

**Figure 1:**
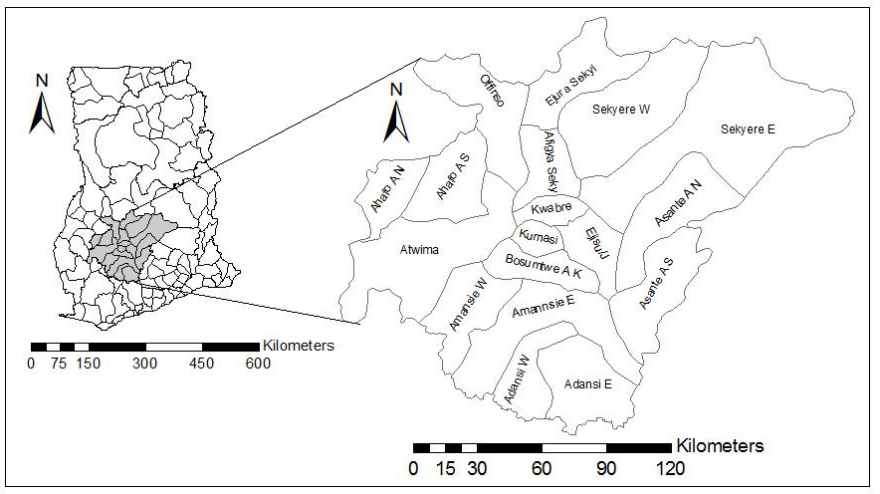
District Map of Ashanti region, Ghana.

The Ashanti Region is centrally located in the middle belt of Ghana. It lies between longitudes 0° 9'W and 2° 15'W, and latitudes 5° 30'N and 7° 27'N. The region shares boundaries with four of the ten political regions, Brong-Ahafo region in the north, Eastern region in the east, Central region in the south and Western region in the South-west (See Figure 1). Ashanti region occupies a total land area of 24,389 km^2 ^representing 10.2% of the total land area of Ghana. Ashanti region has a population density of 148.1 persons per km^2^, compared with a national average of about 80 persons per km^2^. The region consists of 18 administrative districts. Kumasi, which is the capital, is the most populous district, and the only district that has gained a metropolitan status in the region. The 2000 census recorded the region's population as about 3.5 million people, representing 19.1 per cent of the country's population. The urban population (51.3%) in the region exceeds that of the rural population (48.7%). The region is currently the second most urbanized in the country after Greater Accra (87.7%), the national capital. The housing stock in the region is 329,478, of which about 37% are in urban areas and 63% in rural areas. This is in contrast to the 17.4% of houses in urban, and 82.6% in rural areas in 1970. The total stock also represents an increase of 86.8% over the stock in 1984. The relative increase in the proportion of urban housing is a reflection of the increase in urbanization, and perhaps overcrowding. Due to the high housing cost within the urban districts in the region, lots of slummy and/or squatter settlements are created. However, such areas have poor sanitation systems, and perceived to be niches where cholera outbreaks begin.

### Case definition of cholera

In Ghana, a case definition of cholera is based on the WHO's definition which depends on whether or not the presence of cholera has been demonstrated in the area. According to the WHO [41] guidance on formulation of national policy on the control of cholera, in an area where the disease is not known to be present a case of cholera should be suspected, when a patient, 5 years of age or older develops severe dehydration or dies from acute watery diarrhea, or where an epidemic is occurring, a patient, 5 years of age or older develops acute watery diarrhea, with or without vomiting. The first case of cholera however was confirmed by bacteriological tests (personal communication with KMHD director). In this study, only cholera cases made known to the Disease Control Unit (DCU) through reporting facilities such as community volunteers, community clinics, and hospitals were used. In Ghana it is mandatory for all reporting facilities (i.e. hospitals, clinics, and community volunteers) to report weekly cholera cases to the DCU. Although hospitals are scarcely found in many communities in the districts, almost all communities in the districts have access to clinics, and community volunteers who monitor all communicable diseases. The communicable diseases surveillance network is purposely established from community level to district level to ensure effective surveillance of all communicable diseases (personal communication with head of DCU, Ashanti region).

### Spatial data preparation and cartographic display of cholera

Topographic map of the study area at a scale of 1:2500 obtained from the planning unit of the Kumasi Metropolitan Assembly was digitized using ArcGis version 9.2. This software was developed by Environmental System Research Institute (ESRI). Before digitizing, the map was georeferenced (by defining the X and Y coordinates of corner points of the map) into a UTM coordinates system. The main boundary and the 18 districts within the study area were digitized as polygon features. Reported cases of cholera over the period 1997–2001, obtained from the DCU, Ashanti region, were entered as attributes of the districts. The cumulative incidence rates of cholera were calculated for each of the 18 districts by including all cases over the period 1997–2001. The population database was obtained from the 2000 Population and Housing Census of Ghana [42]. This census was conducted by the National Statistical Service of Ghana.

Disease mapping is useful for initial exploration of relationships between exposure and the disease. The raw cumulative incidence rates were smoothed using global Empirical Bayesian Smoothing (EBS) technique. This was to get rid of variance instability as result of heterogeneity in cholera cases and population data (small number problem). The EBS technique consists of computing a weighted average between the raw rate for each district and the regional average, with weights proportional to the underlying population at risk [43]. In effect, districts with relatively small population will tend to have their rates adjusted considerably, whereas for districts with relatively large population the rates will barely change. The resulting smoothed rates were then mapped using GIS. The cutoff points for classification were based on the Jenk's classification technique as employed in ArcGis version 9.2.

### Statistical analyses

In this study, spatial autocorrelation statistic was used to measure the correlation among neighboring observations in a pattern and the levels of spatial clustering among neighboring districts [44,45].

Global Moran's I statistic, which is similar to the Pearson correlation coefficient [46], was calculated as:

$$I=\frac{N}{S_{o}}\sum_{i}\sum_{j}w_{ij}\frac{\left(x_{i}-u)(x_{j}-u\right)}{\sum_{i}{\left(x_{i}-u\right)}^{2}},$$

where *N *is the number of districts, *w*_*ij *_is the element in the spatial weights matrix corresponding to the observation pair *i*, *j*,. Also *x*_*i *_and *x*_*j *_are observations for areas *i *and *j *with mean u.

S_*o *_= ∑_*i*_∑_*j *_*w*_*ij*_

Since the weights were row-standardized ∑*w*_*ij *_= 1. The first step in the analysis of spatial autocorrelation is to construct a spatial weights file that contains information on the neighborhood structure for each location. A first order rook continuity weight file was constructed according to districts who share common boundaries. All spatial autocorrelation analyses were computed using GeoDa 0.9.5-i software [47,43]. A significance test against the null hypothesis of no spatial autocorrelation through a permutation procedure of 999 Monte Carlo replications was used to test for the significance of the statistic. Spatial autocorrelation was calculated for the years 1998, 1999, 2001 and 1997–2001. Because very few cholera cases were reported in 1997 and 2000, autocorrelation was not calculated for these years.

Population based rate ratios were computed for strata of districts grouped by the following variables;

#### Proximity to Kumasi, the most urbanized city

Proximity to Kumasi Metropolis (the most urbanized district in Ashanti region) was categorized into three strata based on the order of spatial neighborhood and/or adjacency. First order neighbors were defined as districts sharing common boundaries with the Kumasi Metropolis. Second order neighbors were defined as districts sharing common boundaries with the first order neighbors, whereas third order neighbors were defined as district sharing common boundaries with the second order neighbors. All spatial neighbors were defined using ArcGIS version 9.2. Population based rate ratios were computed for each strata by taken the stratum of third order neighbors as reference (baseline).

#### Urbanization level

The indicator for urbanization was population based. Each district within the region is made up of localities and/or communities. A locality with a population of 5,000 or more was classified as urban, and less than 5,000 as rural. This is the criteria given by the Ghana Statistical Service [42]. The urbanization level for each district was then computed as the proportion of a district's population residing in localities and/or communities of ≥ 5,000 people in the year 2000. Three urbanization strata were determined, each representing a quartile of districts. Each quartile was composed of six districts. Population-based incidence rate ratios were calculated for each stratum by taking that of lower urbanization as reference.

#### Overcrowding

The indicator for overcrowding was based on four variables: (1) Population density; (2) population per house; (3) single room occupancy (i.e., percentage of households living in single rooms) and; (4) households per house. A household was defined as "a person or group of persons who live together in the same house or compound, sharing the same house-keeping arrangements and are catered for as one unit". Each variable was standardized to have a mean of zero and a standard deviation of one. The variables were combined to form a single index of risk, called overcrowding index (OI). The OI for each district was computed as the mean of the algebraic sum of the standardized values of the four variables. The assumption was that the variables carry equal weights. The Jenk's method of classification was used to classify OI into three strata of districts. Population-based incidence rate ratios were calculated for each stratum by taking that of lower OI as reference (baseline).

Taking into account urbanization stratum and overcrowding level, neighborhood population-based double stratification analyses were performed to explore whether cholera incidence rate was associated with the order of neighborhood with Kumasi Metropolis.

The Extended Mantel-Haenszel *Chi Square *(*χ*^2^) for trend analysis and 95% confidence intervals for rate ratios were computed to explore the relationship between cholera incidence rates and the variables under study [38]. Given a series of proportions representing increasing or decreasing exposure (risk factor) and numbers of affected and non affected people in each stratum (group), the Mantel-Haenszel's extension tests whether the rates in successive groups increase or decrease when compared to the baseline (reference). The results of such test include the rate ratios of successive exposure levels, and *χ*^2 ^and *P*-value (one degree of freedom). A *P*-value less than 0.05 may be taken as reasonable indication of trend in the rates of successive levels compared with the baseline (reference) [EpiInfo version 6.03 help manual]. EpiInfo version 6.03 was used for the computation of the rate ratios, 95% confidence intervals, and the *χ*^2 ^for trend analysis.

## Discussion

This study takes advantage of the advancements in Geographic information system such as spatial disease mapping and smoothing, exploratory spatial data analysis such as spatial autocorrelation, and spatial statistical techniques to identify demographic risk factors of cholera. The extent to which neighboring values are correlated was measured using Global Moran's index for spatial autocorrelation. All autocorrelation analyses suggest significant spatial clustering of cholera with positive Moran's index (see Table 1). This non random distribution also suggests spatial clustering of high rates of cholera incidence at the central part of the region, and low rates at the peripheries (See Figure 2). This is also shown by the high rate ratio of 22 times in the first order neighborhood stratum (i.e. direct neighbors with Kumasi Metropolis, See Table 2). Visual inspections of the EBS maps also suggest possible sustained transmission of cholera at districts within the central part of the region (see Figures 2a, 2b, 2c, 2d). These patterns are plausible largely because of two main reasons. (1) *Demographic status*: Kumasi is the most urbanized and highly commercialized district in Ashanti region, and therefore there is always a high daily influx of traders and civil workers from neighboring districts to Kumasi Metropolis. Such a high daily influx strain existing sanitation systems, thereby putting people at increased risk of cholera transmission. Also, the rural poor most often migrate to city centers with the hope of better life. However, due to the high cost of housing, such migrants settle at slummy and/or squatter areas where environmental sanitation is poor. This largely explains the high *northern population *(inhabitants from the northern sector of Ghana; which is the most deprived sector) within Kumasi Metropolis (2) *Geographic location*: Kumasi Metropolis is the central nodal district of Ghana, and therefore, all road networks linking the northern sector and the southern sector of Ghana pass through Kumasi. There is the high probability of stoppage and transit by travelers, resulting in a high daily population increase and overcrowding at city centers.

This study has also shown that high urbanization and overcrowding are the most important predictors of cholera in Ashanti region, Ghana (See Tables 3, 4, 5, 6). Although cholera is transmitted mainly through contaminated water or food, sanitary conditions in the environment play an important role since the *V. cholerae *bacterium survives and multiplies outside the human body and can spread rapidly where living conditions are overcrowded and water sources unprotected and where there is no safe disposal of solid waste, liquid waste, and human faces [9]. These conditions are met in highly urbanized communities in Ashanti Region. The high rate of urbanization has led to the high level of overcrowding, which necessarily results in shorter disease transmission path. This is shown by the very high rate ratios within the high urban (RR = 19.86) and high OI (RR = 31.11) stratum (See Tables 3 and 4). In fact, the DCU has attributed outbreaks of cholera in urban communities to poor waste management and sanitation systems. In Ghana, urban population growth rate of about 4.3% has outstripped the overall national population growth rate of about 2.7%. The proportion of the population residing in urban areas rose from 32% in 1984 to 43.8% in 2000 [48]. Such high urbanization rate strain existing resources meant for providing better sanitation systems and potable water. Inadequate sanitation systems coupled with intermittent supply of pipe borne water in urban communities puts the population at risk of cholera. Surface water pollution is particularly found to be worse where rivers pass through urban and overcrowded cities, and the commonest contamination is from human excreta and sewage [42]. Due to the cosmopolitan (multi-ethnic) nature of the urban cities in Ghana [49], the traditional laws which were used to protect water bodies form fecal pollution are no longer adhered (Traditionally, it is a taboo to defecate or dispose waste in a water body). Therefore, defecating and dumping of waste in and at the banks of surface water bodies has become a common practice in most urban communities. However, urban inhabitants resort to such polluted water bodies for various household activities like cooking and washing during periods of water shortages.

Further, the rate of slums and/or squatter formation in urban communities is high due to the high rate of migration and population redistribution. Inhabitants living at slums and/or squatter settlements are generally poor, and face problems including access to potable water and sanitation. The urban poor (slums and squatter settlers), are worse off than their rural counterparts in terms of access and affordability to safe drinking water and sanitation. In many cases public utilities providers (e.g. Water distribution) legally fail to serve the urban poor living in slums due to factors regarding land tenure system, technical and service regulations, and city development plans. Most slums and/or squatter settlements are also located at low lying areas susceptible to flooding. Unfavorable topography, soil, and hydro-geological conditions make it difficult to achieve and maintain high sanitation standards among populations living in these territories [19].

This study has shown the capabilities of spatial analysis and GIS in analyzing geographically referenced health data in Ghana. Moreover, the study has also proven that the demographic risk factors of cholera may not be the same in every geographical area or country. For example, Barroto and Martnez-Piedra [19] identified low urbanization as one of the most important ecologic predictors of cholera in Mexico, a Latin American country. However, the results of this study show that high urbanization positively correlates with high cholera incidence.

Although some findings of this research reaffirms the already known hypothesis of cholera, we present the possibility of using GIS and spatial statistical tools for health research in this study area where GIS application in the health sector has not been extensively utilized.

### Limitations of this research

The results of the Extended Mantel-Haenszel *Chi Square *for trend analyses should be interpreted with caution. The number of cholera cases reported to the DCU may only be a fraction of cases that actually occurred, especially in lowly urbanized districts (or rural areas) of the country where level of education is extremely low. It has been suggested that educational level indirectly determines a person's healthcare seeking behavior [18].

The spatial scale of the data may invariably affect the results of the spatial analysis. The areas identified as high risk of cholera are generally large areas defined by administrative boundaries. In such a large spatial scale, it is difficult to demonstrate the actual risk of cholera within a smaller group of people. A more detailed study at a smaller spatial scale is therefore required to assess the accurate individual or smaller groups of individuals' exposure levels. The spatial autocorrelation analysis should be interpreted with caution due the different shapes and sizes of the districts [46].

## Competing interests

The authors declare that they have no competing interests.

## Authors' contributions

FBO carried out the research and drafted the manuscript. AAD guided the research and reviewed the manuscript. All authors read and approved the final manuscript.
